# Evaluation of a Parent Multimedia and Mobile-Based Intervention to Promote Pediatric Oral Health (BeReadyToSmile): Single-Group Pre-Post Feasibility Study

**DOI:** 10.2196/70346

**Published:** 2026-04-07

**Authors:** Erika Westling, David R Smith, Annette Leong, Irin Pimentel-Mannan, Edward G Feil

**Affiliations:** 1 Oregon Research Behavioral Intervention Strategies Inc. DBA Influents Innovations, Springfield, OR, United States; 2 Oregon Research Institute, 3800 Sports Way, Springfield, OR, 97477, United States, +1 541-484-2123

**Keywords:** pediatric oral health, parenting intervention, digital health, mobile health, technology, disparities

## Abstract

**Background:**

The universal adoption of mobile technologies by households has created an opportunity to provide families with young children with access to high-quality oral health information at convenient times and locations. Using community agencies (eg, Head Start and public health programs) that offer parenting education is an effective approach to reaching families in low-income households.

**Objective:**

This study aimed to explore the extent to which a coordinated, in-person oral health prevention intervention, together with an accompanying smartphone app, BeReadyToSmile, is feasible to implement among caregivers of young children.

**Methods:**

The BeReadyToSmile program targeted parents of children aged 0 to 6 years attending parenting education classes. This study was designed as a single-group pre-post feasibility study that included quantitative surveys and open-ended feedback. A total of 30 parents attended an in-person session on child oral health and were invited to use the BeReadyToSmile smartphone app. Preintervention and postintervention surveys were administered to assess pediatric oral health knowledge, attitudes toward child toothbrushing, brushing intention, brushing efficacy, program satisfaction, and ease of use.

**Results:**

Significant effects were observed on parent-reported pediatric oral health knowledge, attitudes toward brushing, brushing intention, and toothbrushing efficacy. Out of the 30 parents invited to use the BeReadyToSmile app, 1 (3%) completed no sessions and 20 (67%) completed all sessions. Participants rated the app highly on measures of satisfaction and use. We found significant increases in pediatric oral health knowledge (*P=*.004), child brushing attitudes and intention (*P*=.01), and parental efficacy regarding child toothbrushing (*P*=.03).

**Conclusions:**

Caregivers reported positive experiences with the implementation of BeReadyToSmile, indicating the overall feasibility of delivering oral health prevention to households with young children both in person and through a facilitated smartphone app. Further studies should include a larger and more diverse sample, randomized comparison conditions, and a longer follow-up period to assess outcomes.

## Introduction

### Background

Dental caries (tooth decay) is a highly prevalent chronic disease affecting a significant proportion of the US population, including young children [1]. Lower-income children are especially at risk for dental caries, and this disparity by income has been consistent for decades [1,2]. Oral disease poses a significant risk to overall health throughout one’s life [3-8], although dental caries is a preventable disease that can be stopped and even potentially reversed during its early stages [9]. The American Dental Association, the American Academy of Pediatric Dentistry, and the American Academy of Pediatrics recommend fluoride toothpaste for all children, as twice-daily toothbrushing with fluoridated toothpaste is widely endorsed by dental associations as a key preventive measure against dental caries [10-12]. However, most families do not adhere to these recommendations, with nearly 80% of children starting toothbrushing later than recommended and almost one-third brushing just once daily [13]. For children aged less than 7 years, there can be significant barriers, including parents’ oral health beliefs, social norms, children’s emotional reactions, and a lack of parenting skills [14].

Research indicates that parent education can be effective in improving children’s toothbrushing practices and overall oral hygiene. When accompanied by social support and in-person instruction, toothbrushing promotion programs have reduced childhood caries and improved oral hygiene [14-20]. A randomized trial showed that children whose mothers received repeated rounds of oral health education experienced a significant decrease in the incidence of early childhood caries [21]. These findings emphasize the importance of providing oral health education and support to parents, particularly those from disadvantaged backgrounds, to enhance children’s oral health outcomes.

One barrier to providing pediatric oral health promotion with parenting support is the inaccessibility of services for families, particularly for those who live in rural areas and for racial and ethnic minority populations. Digital or telehealth service delivery has gained significant traction in the medical field and was further accelerated during the COVID-19 pandemic [22], including teledentistry [23,24]. Pediatric dental education and behavioral interventions have been identified as promising applications of mobile health technology in the post–COVID-19 era [23,24]. In addition, there is a growing infrastructure for delivering parenting education and support via parenting classes [25], with an emphasis on reaching underserved populations. For example, in Oregon, where this study took place, the Oregon Parenting Education Collaborative (OPEC) has made significant headway in providing parenting education programs to previously underserved populations. Since 2010, OPEC has served 14,207 Oregon parents through evidence-based classes, 14,504 families through home visits, and more than 950,000 family members through parenting education activities [26]. Moreover, programs such as Head Start and Early Head Start have recognized the importance of dental health, incorporating it into child health standards [27-30].

Despite research demonstrating the positive impact of parent education on reducing childhood caries and promoting oral health behaviors, current parenting education curricula in programs such as Head Start do not adequately address children’s dental health education. Most significantly, available programs are instructional in nature. They do not demonstrate evidence-based parenting skills across various age groups, particularly when faced with a child’s resistance to essential dental hygiene routines. Many of the best empirically based parenting interventions start with positively oriented strategies and incorporate behavioral parent training [31,32], including Parent-Child Interaction Therapy [33], the Positive Parenting Program (Triple P [34]), and the Incredible Years programs [35]. These effective intervention approaches prioritize reinforcing appropriate and desired behaviors as the primary catalyst for improving child behavior; however, these approaches are not currently integrated into pediatric oral health education offered through Head Start and other parenting programs.

Thus, while parent-focused education and support can improve children’s oral hygiene and reduce caries, families most at risk, including those in rural communities and those with lower incomes, often do not have access to in-person services. At the same time, widely used parenting and early childhood support programs do not typically provide skill-based, evidence-informed behavioral parenting strategies tailored to oral hygiene challenges. Existing content is often instructional but does not provide caregivers with the tools to respond effectively when children resist daily dental hygiene routines. Consequently, there is a need for an accessible, scalable pediatric oral health promotion program with digital delivery that integrates evidence-based behavioral parent training with established oral health guidance to help caregivers build effective routines and reduce child resistance, ultimately improving children’s oral health outcomes and narrowing disparities. BeReadyToSmile was developed to be such a program, integrating empirically supported behavioral parent training with established best practices for pediatric oral health within a comprehensive, universal oral health education program.

### Objectives

This feasibility study examines the practicality of implementing BeReadyToSmile in real-world settings, with a focus on caregiver knowledge, confidence, and efficacy regarding child oral health behaviors; program reach and engagement; perceived program satisfaction and usability; delivery fidelity; and logistical barriers, such as access to technology.

## Methods

### Ethical Considerations

This study was registered as a clinical trial at ClinicalTrials.gov (NCT03637309) on September 8, 2017. The Oregon Research Institute’s Institutional Review Board approved this study (082817) on August 28, 2017. This study was deemed to pose no more than minimal risk. All participants provided informed consent before engaging in any study activities. To protect participants’ confidentiality and privacy, all data were deidentified and accessible only to the research team, and all published data are presented in aggregate form. All data are securely stored and will be destroyed within 5 years of publication. Participants received US $30 per data collection session.

### Participants

Participants were parents or caregivers recruited from Head Start of Lane County and OPEC. For this pilot study, the program included 2 video sessions viewed in a group setting over 2 parenting classes and a smartphone app with dental educator–facilitated goal setting and feedback. Data were collected from 30 consenting parents or caregivers participating in a group-based parent education program who also volunteered to use the BeReadyToSmile app.

### Program Development

The BeReadyToSmile development process was iterative, incorporating parents’ feedback and program revisions throughout development. In the BeReadyToSmile program, parents teaching their children oral health behaviors, such as toothbrushing, are seen as opportunities for children to learn critical health-preserving skills and for parents to strengthen their parenting skills. Research staff conducted focus groups with parents of young children in educational groups. We presented the American Dental Association video “Your Child’s Teeth: Birth to Age 5” [36]. Parents completed surveys to provide feedback on the video. This information enabled us to create realistic video vignettes and strategies that were tailored to the life experiences of the target users, a key component in developing an intervention for self-learning. The final 30-minute video consists of 7 segments using a variety of approaches, including parent group discussions, animation, and direct informational content. The segments cover (1) the importance of children’s teeth and dental health, (2) giving only water in bottles at bedtime, (3) the value of routine and positive reinforcement, (4) daily toothbrushing with fluoride, (5) a “Sugar Shocker” segment, (6) a conversation about fluoride, and (7) a recap and review.

**Figure 1.**
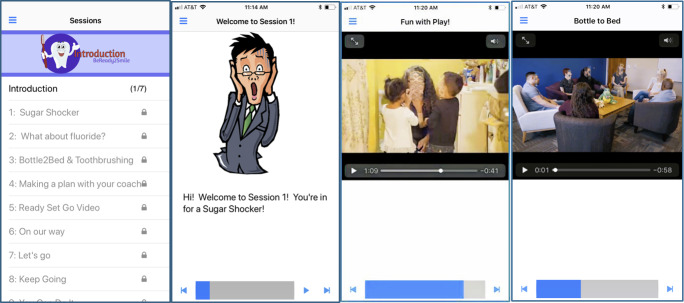
Screenshots from the smartphone app depicting (left to right) the topics to be covered, the beginning of Sugar Shocker, a video demonstrating how to engage young children in toothbrushing, and a video of a group discussion about using water in children’s nighttime bottles.

The BeReadyToSmile in-person video session used videos developed for the smartphone app (Table 1). The BeReadyToSmile mobile app was designed for both iOS and Android operating systems, as well as a web browser, with three key components: (1) a self-regulated course for learning parenting skills with dynamic multimedia presentations and interactive queries, (2) an electronic messaging system for coaching support to facilitate learning and promote engagement, and (3) an online tracking system of participant knowledge acquisition and intervention engagement to facilitate progress monitoring and supervision. BeReadyToSmile was designed as a series of sequential sessions, so that before the user could proceed to the next session, the previous session must be complete. Users could return and review previously completed material in a nonlinear format. The software uses a menu-driven approach (Figure 1). Video and audio narration were used wherever possible to minimize typing skills and literacy requirements. Users could (1) view session content, (2) upload videos or images of parent-child toothbrushing, (3) view links to dental resources, (4) send a message to their coach, (5) communicate with their coach to develop an action plan and set goals, (6) view the action plan developed with their coach, (7) earn stars and badges for completing sessions or reaching goals, (8) change settings such as their password, and (9) log out of the app.

**Table 1.** Overview of the BeReadyToSmile program, delivered both in-person and via a facilitated smartphone app.

Program component and sessions	Description	Topic or activities
Videos shown during in-person parent group		
Session 1	Overview of child dental health recommendations	Importance of children’s teeth and dental health Only water in bottles at bedtime Value of routine and reinforcement Daily toothbrushing with ﬂuoride
Session 2	Reduction of sugar consumption and use of fluoride	Sugar Shocker Fluoride conversation Review
Smartphone app		
Session 3	Reinforcement of videos shown in sessions 1 and 2	Introduction Topic 1: Sugar Shocker Topic 2: all about fluoride Topic 3: Bottle2Bed and toothbrushing
Session 4	Motivational interview with a coach to create a dental behavior change plan tailored to the participant and their family	Topic 4: making a plan with your coach
Session 5	Individualized support to implement tailored plans through parent-child video, badges and stars, and coach messaging	Topic 5: ready-set-go Topic 6: on our way Topic 7: let’s go Topic 8: you can do it Topic 9: keep going Topic 10: you did it!

### Data Collection

This single-group preintervention and postintervention study assessed parent or caregiver knowledge, attitudes, and confidence in promoting their young child’s oral health, as well as their satisfaction with, and the usability of, the BeReadyToSmile mobile app. Data collection occurred across 4 weeks. Of the 30 parents, 25 (83%) completed the preintervention survey, 17 (57%) completed both the preintervention and postintervention surveys, and an additional 4 (13%) participants completed the postintervention survey but not the preintervention survey.

The preintervention survey included demographic information and technology use, specifically internet access, mobile device use, and social media use. At preintervention and postintervention surveys, participants completed a 15-item knowledge inventory of young children’s oral health [37], including recommendations for home hygiene and dental health (9 items), statements about the caries process (3 items), and dental development (3 items). For each item, participants indicated their level of knowledge on a three-point scale: (1) “didn’t know,” (2) “sort of know,” and (3) “know for sure.” The knowledge inventory was validated in a randomized controlled trial of a parent-focused intervention that significantly improved twice-daily toothbrushing among young children [37].

Participants also completed scales assessing their attitudes toward child oral health promotion: the “Importance and Intention to Brush Child’s Teeth” scale (5 items) and the “Parental Efficacy in Relation to Child Toothbrushing” scale (6 items) [38]. These measures, developed by Adair et al [38], have been validated through international research on pediatric dental health. Response options were measured on a 5-point Likert scale, ranging from “strongly disagree” (1) to “strongly agree” (5) for the importance and intention items and from “strongly agree” (1) to “strongly disagree” (5) for the parental efficacy items. Participants also reported their confidence in brushing their young child’s teeth, responding to the question: “If you already don’t brush twice a day, how confident are you that, if you decided to, you could brush your child’s teeth twice (or almost always twice) a day?” Responses were recorded using a 10-point Likert scale ranging from “not confident at all” (1) to “very confident” (10).

At the postintervention survey, participants rated their satisfaction and ease of use with either the in-person video session (T2) or the BeReadyToSmile app (T4) on a 4-point Likert scale ranging from “not at all” (negative; 1) to “very” (positive; 4). Questions included “How reasonable did you find the app?” “How much did you like the app for learning the information taught?” “How clear was the information taught?” “How useful did you find BeReadyToSmile?” and “I would recommend the BeReadyToSmile app to other parents.” During app use, the software recorded participant activity in a MySQL database. The use included the following indices: (1) attrition, (2) session completion, and (3) extent of intervention participation (eg, number of app topics completed).

## Results

### Participant Demographics

Out of the 30 participants, 25 (83%) completed the demographic items in the preintervention survey. Parents’ or caregivers’ mean age was 36.64 (SD 8.18) years; 4 (16%) were male, 19 (76%) were White, and 5 (20%) were Hispanic. In total, 44% (10/23) reported working full time, but 48% (11/23) reported some or a great deal of difficulty paying monthly bills, and 57% (13/23) had a combined annual household income of US $30,000 or less. All families (N=25, 100%) reported using phones for internet access. Table 2 shows additional details.

**Table 2.** Participant demographics.

Variable	Values
Age (years), mean (SD)	
Children (n=24)	5.34 (2.46)
Parents (n=25)	36.64 (8.18)
Number of participants living at home, mean (SD)	
Adults (n=25)	2.28 (1.02)
Children (n=24)	2.21 (1.14)
Parents living together or married (n=25), n (%)	15 (60)
Ethnic groups (n=25), n (%)	
Hispanic	5 (20)
White	19 (76)
Household socioeconomic characteristics, n (%)	
Parents with educational attainment of high school or less (n=25)	10 (40)
Parent in household working full time (n=23)	10 (44)
Parents reported “some” or “a great deal” of difficulty paying monthly bills (n=23)	11 (48)
Combined annual household income of US $30,000 or less (n=23)	13 (57)

### Outcomes on Knowledge, Attitudes Toward the Importance of Brushing, and Confidence

A total of 17 participants completed both the preintervention and postintervention surveys. Using 1-tailed *t* tests, there were significant effects from the preintervention survey to the postintervention survey on knowledge (*t*_16_=3.35; Cohen *d*=0.81; *P*=.004) [37], attitudes toward the importance of toothbrushing and brushing intention (*t*_16_=2.75; Cohen *d*=0.66; *P*=.01) [38], and toothbrushing efficacy (*t*_16_=2.41; Cohen *d*=0.59; *P*=.03) [38]. Confidence approached statistical significance (*t*_16_=2.09; Cohen *d*=0.51; *P*=.05) [37]. Refer to Table 3 for pre-post outcomes. In addition, 81% (17/21) of parents reported an increase in their child’s cooperation following participation in the BeReadyToSmile program.

**Table 3.** Preintervention survey to postintervention survey outcomes (N=17)^a^.

Variable	Preintervention survey, mean (SD)	Postintervention survey, mean (SD)	*t* test (*df*)	*P* value
Knowledge inventory	31.82 (8.03)	39.12 (6.19)	–3.35 (16)	.004
Importance of toothbrushing and intention to brush your child’s teeth	4.22 (0.56)	4.60 (0.45)	–2.75 (16)	.01
Parental efficacy in relation to child toothbrushing	1.82 (0.62)	1.53 (0.51)	2.42 (16)	.03
Parental confidence	7.65 (2.65)	9.06 (1.75)	–2.09 (16)	.05

a Response options for the 15-item knowledge inventory were 1‐3, with 1 being “didn’t know,” 2 being “sort of know,” and 3 being “know for sure”; a higher summed score indicates a higher level of knowledge [37]. Response options for the 5 importance or intention items were measured on 5-point Likert scales ranging from 1 (“strongly disagree”) to 5 (“strongly agree”); higher mean scores indicate greater perceived importance and stronger intention to brush a child’s teeth [38]. Response options for the 6 parental efficacy items were also measured on 5-point Likert scales but ranged from 1 (“strongly agree”) to 5 (“strongly disagree”); lower mean scores indicate greater parental efficacy. Parental confidence was assessed using a 10-point Likert scale ranging from 1 (“not confident at all”) to 10 (“very confident”) in response to the following item: “If you do not already brush twice a day, how confident are you that, if you decided to, you could brush your child’s teeth twice (or almost always twice) a day?” Higher scores indicate greater confidence.

### Satisfaction and Usability

Mean ratings were above 3.3 on a 4-point scale (Table 4). Caregiver comments on open-ended postintervention survey items regarding their satisfaction with, and perceived usability of, the app included the following: “I really liked using the mobile app,” “I enjoyed the break-down of the videos/sessions,” “It’s nice to hear something positive for a change,” “Making it fun by having them brush my teeth, too. I feel it will help with their ‘no,’ and I’m going to aim for 2x a day now,” and “Brushing together as a family, and making a game out of brushing teeth.”

**Table 4.** Participant satisfaction and usability ratings (N=21)^a^.

BeReadyToSmile app satisfaction and usability evaluation items	Satisfaction and usability rating, mean (SD)
How reasonable did you find the BeReadyToSmile app for learning information taught?	3.48 (0.68)
How much did you like the BeReadyToSmile app for learning the information taught?	3.48 (0.60)
How clear was your understanding of the BeReadyToSmile app information taught?	3.67 (0.58)
How useful did you find the BeReadyToSmile app in addressing questions about a child’s dental care?	3.57 (0.60)
My relationship with my coach was very important to me.	3.38 (0.67)
I would recommend the BeReadyToSmile app to other parents.	3.57 (0.60)

a Response scales for the first 5 items were 1‐4, with 1 being “not at all” and 4 being “very.” The response scale for the final item was 1‐4, with 1 being “not at all recommend” and 4 being “strongly recommend.” Higher scores indicate higher satisfaction and usability.

### BeReadyToSmile App Use

Participants actively used the app during the research period. Across the 4 weeks of testing, participating parents or caregivers spent an average of 2.63 (SD 0.76) hours using the app and completed an average of 8.33 (SD 3.99) sessions. Table 5 shows the number of sessions completed, indicating a high level of engagement, with most participants completing all 10 sessions. Between parents and the coach, 1127 text messages were exchanged. We did not find a significant relationship between the number of hours, sessions, or topics completed and outcomes, possibly due to a ceiling effect with a restricted range.

**Table 5.** Number of app topics completed by participants (N=30)^a^.

Number of app topics completed	Participants, n (%)
0	1 (3)
1	2 (7)
2	2 (7)
4	1 (3)
5	4 (13)
10	20 (67)

a There were 10 possible topics to complete in the app.

## Discussion

### Principal Findings

The BeReadyToSmile pilot program demonstrated promising results in addressing a critical gap in pediatric oral health education for low-income families. As anticipated, the intervention had a significant positive impact on parental knowledge, attitudes, and self-efficacy regarding their children’s oral health, echoing findings from previous studies that have emphasized the importance of education in preventing childhood dental caries [39,40]. The improvement in knowledge and attitudes among participants from the preintervention to the postintervention survey aligns with findings that education, when paired with personalized feedback and support, can effectively influence oral health behaviors [41].

A critical challenge this program addresses is the dearth of oral health resources that are both relevant and engaging for parents from diverse, low-income backgrounds. This study reinforces the need for culturally tailored interventions that address the unique barriers faced by these populations, including language differences, health literacy challenges, and culturally specific beliefs about oral health [39]. The success of the BeReadyToSmile program in integrating parents’ real-life experiences into its design, as reflected in the video content and interactive app features, serves as an important model for future interventions aiming to bridge these gaps.

The high satisfaction rates reported by participants, along with the observed increases in child cooperation during toothbrushing, underscore the value of engaging, family-centered approaches to oral health promotion. Parent comments indicated that making toothbrushing a fun, collaborative activity positively impacted their children’s behavior and improved consistency in daily toothbrushing routines. These findings are consistent with prior research suggesting that interventions that focus on building positive parent-child interactions around health behaviors can lead to lasting improvements in health outcomes [42].

Importantly, the high level of engagement with the BeReadyToSmile app suggests strong feasibility and acceptability. Approximately two-thirds of participants (20/30, 67%) completed all 10 app topics, and more than 1100 text messages were exchanged between parents and coaches, reflecting the importance participants placed on both the self-guided and interactive coaching components. As the results suggest, digital interventions can overcome barriers such as geographic distance and time constraints [42]. However, it is likely that this restricted range of high engagement did not result in a significant relationship between engagement and outcomes due to a ceiling effect.

### Strengths and Limitations

One strength of the BeReadyToSmile program is its use of digital technology to increase access to oral health education. Because most families in this study already used smartphones as their primary source of internet access, the app’s technology requirements were minimal, making it well suited for embedding into community-based programming without significant infrastructure changes. Thus, another strength is the program’s demonstrated potential for integration into existing parent education and early childhood frameworks, such as Head Start, Early Head Start, and OPEC.

Despite the positive outcomes, several challenges were noted. First, this pilot study involved a small sample size, limiting the generalizability of the findings. A second limitation of the BeReadyToSmile program itself is its reliance on facilitated, coach-supported engagement, which requires substantial staffing resources. This limitation highlights the need for future iterations of the program to explore more scalable, self-directed formats that maintain the intervention’s effectiveness while reducing staff burden. One possible approach may be integrating artificial intelligence into the coaching. Third, while the single-group design provided valuable preliminary data, future research should include a randomized controlled trial with longer-term follow-up to rigorously evaluate the program’s impact and compare it with traditional oral health education methods. Objective measures of oral health outcomes, such as clinical assessments of caries incidence, would also strengthen future research. Additionally, evaluating the program’s cost-effectiveness and its impact when implemented at scale could inform policy and funding decisions.

### Conclusions

The BeReadyToSmile program shows promise as an innovative approach to pediatric oral health promotion, integrating accessible technology, evidence-based parenting strategies, and culturally relevant content to address disparities in early childhood caries. Findings from this pilot suggest that digital, parent-focused oral health education can effectively enhance oral health knowledge and behaviors among low-income families, particularly when paired with parenting support. Further research with larger, more diverse samples is needed to refine and confirm the model’s effectiveness, but expanding access through partnerships with early childhood services could significantly improve oral health outcomes for underserved populations.

## Abbreviations

OPEC: Oregon Parenting Education Collaborative
